# BYPASS1: synthesis of the mobile root-derived signal requires active root growth and arrests early leaf development

**DOI:** 10.1186/1471-2229-11-28

**Published:** 2011-02-03

**Authors:** Jaimie M Van Norman, Caroline Murphy, Leslie E Sieburth

**Affiliations:** 1 Department of Biology, University of Utah, 257 South 1400 East, Salt Lake City, Utah, 84112, USA; 2 Biology Department, Duke University, Durham, North Carolina, 27708, USA

## Abstract

**Background:**

The Arabidopsis *bypass1 *(*bps1*) mutant root produces a biologically active mobile compound that induces shoot growth arrest. However it is unknown whether the root retains the capacity to synthesize the mobile compound, or if only shoots of young seedlings are sensitive. It is also unknown how this compound induces arrest of shoot growth. This study investigated both of these questions using genetic, inhibitor, reporter gene, and morphological approaches.

**Results:**

Production of the *bps1 *root-synthesized mobile compound was found to require active root growth. Inhibition of postembryonic root growth, by depleting glutathione either genetically or chemically, allowed seedlings to escape shoot arrest. However, the treatments were not completely effective, as the first leaf pair remained radialized, but elongated. This result indicated that the embryonic root transiently synthesized a small amount of the mobile substance. In addition, providing glutathione later in vegetative development caused shoot growth arrest to be reinstated, revealing that these late-arising roots were still capable of producing the mobile substance, and that the older vegetative leaves were still responsive.

To gain insight into how leaf development responds to the mobile signal, leaf development was followed morphologically and using the CYCB1,1::GUS marker for G2/M phase cells. We found that arrest of leaf growth is a fully penetrant phenotype, and a dramatic decrease in G2/M phase cells was coincident with arrest. Analyses of stress phenotypes found that late in development, *bps1 *cotyledons produced necrotic lesions, however neither hydrogen peroxide nor superoxide were abundant as leaves underwent arrest.

**Conclusions:**

*bps1 *roots appear to require active growth in order to produce the mobile *bps1 *signal, but the potential for this compound's synthesis is present both early and late during vegetative development. This prolonged capacity to synthesize and respond to the mobile compound is consistent with a possible role for the mobile compound in linking shoot growth to subterranean conditions. The specific growth-related responses in the shoot indicated that the mobile substance prevents full activation of cell division in leaves, although whether cell division is a direct response remains to be determined.

## Background

Plants synthesize a wide array of metabolites, and a major goal of metabolomics is to identify natural plant metabolites and their associated functions (reviewed in [1-3]). Recent advances facilitating identification of metabolites [4,5] have led to identification of groups of metabolites that correlate with important plant traits, such as growth rate and biomass [6,7], and identified metabolic regulators such as leucine [8]. However, how specific metabolites other than characterized hormones function in signaling and development is largely unknown. One approach to learning about alternate signaling molecules is to study mutants with signaling-related defects.

The Arabidopsis *bypass1 *(*bps1*) mutant might be an important tool for identifying a metabolite functioning as a long-distance signal. The *bps1 *mutant produces small abnormal roots and shoot development arrests soon after germination. This phenotype is linked to a mobile substance as the *bps1 *mutant root is necessary to induce arrest of *bps1 *shoots, and in graft chimeras, the *bps1 *root is sufficient to induce arrest of the wild-type shoot [9]. These observations led to a model featuring BPS1 as a negative regulator that was required to prevent the excess production of a mobile substance. The mobile compound appears to be novel, and its synthesis requires carotenoid biosynthesis [10]. The pathway producing the *bps1 *mobile compound appears to be conserved in plant lineages, as knock-downs of conserved *BPS-*like genes in tobacco produced similar phenotypes [11]. Critical questions include whether this mobile compound is an endogenous developmental regulator, and how it modifies shoot growth.

Control over shoot branching by a root-derived signal has been elegantly analyzed in pea, rice, and Arabidopsis [12-15]. In these systems, mutations disrupting biosynthetic enzymes lead to reduced production of a mobile compound that controls auxin transport in the shoot [16,17]. Recently, this substance was identified as strigolactone [18,19]. Additional unknown root-to-shoot signals have been implicated by studies of drought (reviewed in [20]), soil compaction [21], nutrient depletion [22-24] and low-fluence UV-B light [25]. The identities of the mobile compounds elicited by these treatments are unknown; it is also unknown whether the *bps1 *mobile substance is related to any of these pathways, but its root-to-shoot mobility make it an attractive candidate.

It is also possible that the *bps1 *mobile compound could instead be an intermediate molecule that normally doesn't accumulate. For example, a biosynthetic pathway might be blocked in *bps1 *mutants, resulting in build-up of a precursor that happens to be mobile, and happens to have biological activity. For example, in *superroot1 *mutants, a defect in glucosinolate biosynthesis causes a build-up of precursors that spills over into auxin biosynthesis, resulting in a high-auxin phenotype [26].

Here, we evaluate the conditions under which *bps1 *roots produce the mobile compound, and the characteristics of shoots undergoing arrest from this substance. We find that *bps1 *roots produce and transport the mobile substance in actively growing roots, but that arrest of cell division leads to cessation of signaling to the shoot. Shoot responses include growth cessation, and in particular, arrest of cell division.

## Results

### The *bps1 *root: shoot growth inhibition requires root growth

The central feature of *bps1 *mutants is that a growth-arresting mobile compound arises in the root [9]. However, the experimental basis for this assignment required wounding, and it was only tested in very young seedlings. To expand our understanding of the root's role in producing the *bps1 *signal, we examined how leaf development responded when *bps1 *root growth and development was blocked after embryogenesis. Post-embryonic root growth and development requires glutathione (GSH, [27]). Root development can therefore be blocked by either supplying germinating seeds with L-buthionine sulfoximine (BSO), an inhibitor of γ-glutamylcysteine synthetase, or by generating double mutants between *bps1 *and *root meristemless1-1 *(*rml1-1*), which has a defect in the gene encoding γ-glutamylcysteine synthetase and lacks post-embryonic root development [27,28].

**Figure 1:**
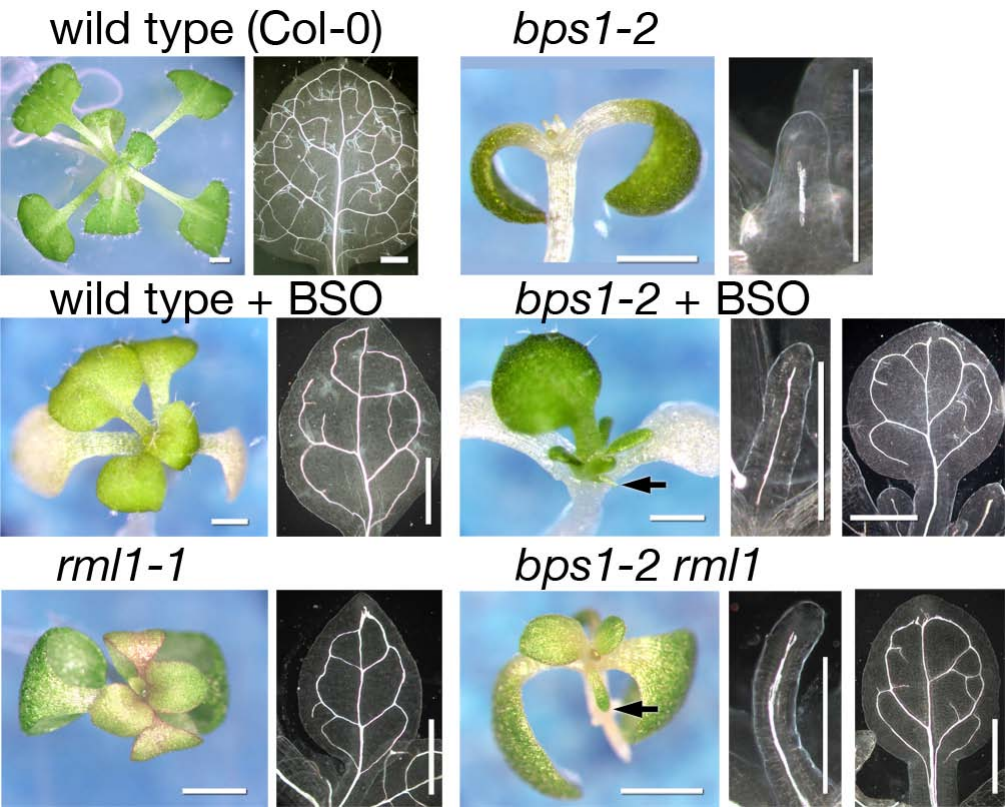
**Sustained synthesis of the *bps1 *mobile compound requires post-embryonic root growth**. The top row shows wild type seedlings with their broad flat leaves and highly interconnected leaf veins, and *bps1-2 *mutants, with their very small radial leaf primordia and incomplete primary vein. The second row shows the effect of BSO-induced root arrest on wild type and *bps1 *mutants. In the wild type, shoots of BSO-grown plants had chlorotic cotyledons and small leaves with modestly reduced vein patterns. The shoots of *bps1-2 *mutants growth on BSO-containing media produced two types of leaves: the first leaf pair (black arrow) were elongated but radial, and contained only a single vein, while leaves that arose later were broad, flat, and produced more complex vein patterns. The third row shows *rml1-1 *and *bps1-2 rml1-1 *double mutants. The *rml1-1 *mutants were slightly smaller than the wild type and they produced slightly narrow, pointed leaves with modestly reduced patterns of veins. The *bps1-2 rml1-1 *double mutants produced two types of leaves: the first leaf pair (black arrow) were radialized but elongated, and contained a single vein, while later leaves were broad, flat, and produced complex vein patterns. Size bars: 500 μm.

In agreement with previous publications, the roots of BSO-treated wild type appeared to arrest development at germination. In addition, these plants produced small, but flattened, leaves with distinct blade and petiole, a modest reduction in leaf vein pattern, and bleached cotyledons (Figure 1). By contrast, *bps1 *mutants grown on BSO-supplemented medium produced leaves with two distinct shapes (Figure 1). The first leaf pair was small, radially symmetric, and contained only a single unbranched vein, while subsequently produced leaves were broad, flat, showed distinct blade and petiole, and contained both primary and secondary veins. This partial rescue of leaf development in BSO-treated *bps1 *mutants suggested that post-germination arrest of the *bps1 *root led to reduced synthesis of the root mobile signal.

Similarly, the roots of *rml1-1 *mutants appeared to arrest development at germination, and they produced small, flattened leaves with distinct blade and petiole (Figure 1). Growth of *rml1-1 *mutants on GSH-supplemented medium restored post-embryonic root growth, as reported previously [27] (Table 1). The *bps1 *single mutants and F2 seedlings derived from *rml1-1*/+ *bps1-2*/+ parents, grown on GSH-supplemented media, were indistinguishable from *bps1 *controls (Table 1). By contrast, F2 seedlings derived from *rml1-1*/+ *bps1-2*/+ parents grown on standard growth medium (lacking GSH), segregated for four different phenotypes: wild type; *rml1-1*; *bps1-2*; and a phenotype similar to BSO-grown *bps1 *mutants (Figure 1). This last phenotype appeared at numbers consistent with it being the *rml1-1 bps1-2 *double mutant (Table 1). As with *rml1-1*, the *bps1 rml1-1 *double mutants produced roots that showed no sign of post-embryonic cell divisions. However, their first pair of leaves were radial and contained a single unbranched vein; these were similar to the *bps1 *single mutant, but much longer (Figure 1). Strikingly, leaf 3 and subsequently produced leaves were broad and flattened, with distinct petiole and blade, and contained both primary and secondary veins, much like the leaves of *rml1-1 *single mutants and BSO-treated *bps1 *(Figure 1). Thus, these data indicate that post-embryonic root growth and development is required for continuous production and delivery of the leaf-arresting substance and further support the root as the source of this molecule [9].

**Table 1:** The *bps1 *shoot phenotype requires post-embryonic root development

			Shoot Phenotypes Observed				
				
Plants analyzed	GSH	Total (n)	wild type (n)	*rml1 * (n)	*bps1 * (n)	*bps1 rml1 * (n)	χ^2^
*rml1-1*	-	240	189	51	0	0	1.8^a^
*rml1-1*	+	70	70	0	0	0	n/a
*bps1-2*	-	224	164	0	60	0	0.381^a^
*bps1-2*	+	111	85	0	26	0	0.147^a^
*rml1-1 bps1-2 *F2	-	1258	728	236	210	84	3.793^b^
*rml1-1 bps1-2 *F2	+	590	437	0	153	0	0.2734^a^

n/a, not applicable

^a ^Critcal χ^2 ^values at 95% confidence is 3.841 when *df *= 1.

^b ^Critical χ^2 ^values at 95% confidence is 7.815 when *df *= 3.

Because imposing a GSH deficit (using either BSO or *rml1-1*) led to partial rescue of the *bps1 *shoot phenotype, it was a formal possibility that the *bps1 *root-derived compound could be GSH itself. To test this, we supplied GSH to excised *bps1 *shoots, and monitored subsequent leaf development. Typically, root excision leads to partially rescued shoot development in approximately 75% of *bps1 *single mutants [9]. We reasoned that if the mobile signal was GSH, supplying it to *bps1 *mutants following root excision would reduce the number of *bps1 *mutants that were rescued by root excision. However, supplying GSH did not diminish shoot developmental rescue, and no regimen of GSH provision (prior to cut, after cut, or both prior and after cut) yielded a statistically significant reduction of developmental rescue. Moreover, shoot rescue resulted in leaves reaching similar sizes, whether or not excised shoots were supplemented with GSH (Table 2). These data indicated that the mobile compound was not GSH.

**Table 2:** Analysis of GSH as a candidate for the BPS1-regulated signal

Growth Medium		Total *bps1 *with excised root (n)	Percent producing broad leaves with distinct blade and petiole (n)
Pre-excision	Post-excision		
GSH-	GSH-	31	77% (24)
GSH-	GSH+	40	83% (33)
GSH+	GSH-	27	70% (19)
GSH+	GSH+	34	94% (32) **

** The number of plants that produce leaves under GSH+/GSH+ conditions is statistically greater than the number that produces leaves under GSH-/GSH-conditions (p value = 0.009).

### Arrested roots provide a transient source of the *bps1 *signal

Arrest of post-embryonic root growth in *bps1 *caused strikingly different responses in the first leaf pair as compared to leaf three. Both BSO-grown *bps1 *mutants and *rml1 bps1 *double mutants initially produced a pair of radialized leaves, yet rescued development was observed in subsequently produced leaves (Figure 1). In addition, the *rml1-1 bps1-2 *first leaf pair was consistently larger than that of BSO-grown *bps1-2 *mutants. These results contrast to root excision (carried out at day 4), where the strongest rescue was observed in the first leaf pair [9], and suggest that the arrested *bps1 *root might be a transient source of the *bps1 *mobile compound.

We tested this possibility using the *bps1 *temperature dependent phenotype [9]. We compared the first leaf pair of *bps1 rml1-1 *double mutants grown at 16, 22, and 29°C. For *bps1 *single mutants, leaf development is temperature dependent: severe arrest occurs at low temperatures, and small but flattened leaves are produced at high temperatures. Thus, if the radialized first leaf pair of *bps1 rml1-1 *double mutants was due to the *bps1 *mobile signal, then we expected its development to be similarly dependent on growth temperature.

**Figure 2:**
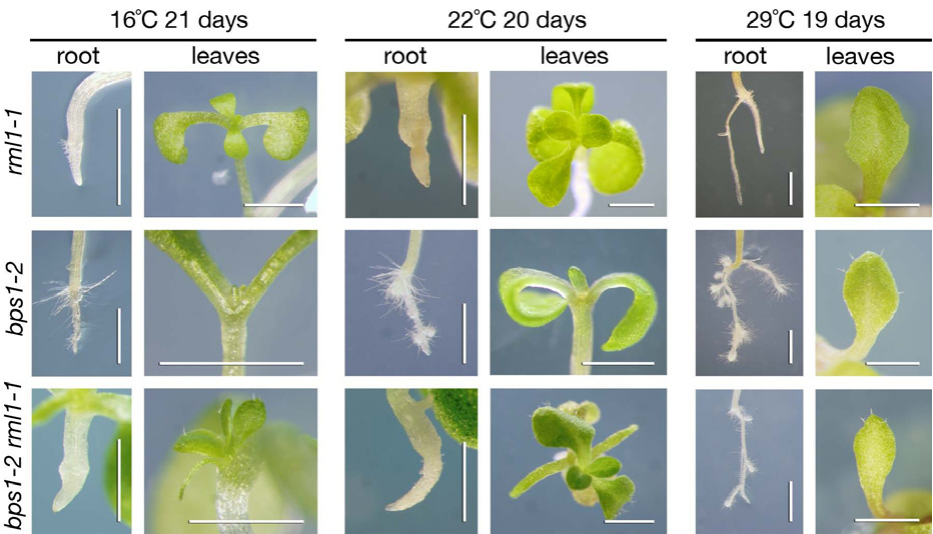
**Temperature responsive phenotype of the *bps1 rml1 *first leaf pair supports exposure to the *bps1 *mobile compound during development**. In *bps1 *single mutants, leaf development arrests early at low temperature (16°C), while growth at higher temperatures results in progressively more leaf growth. In *bps1-2 rml1-1 *double mutants, development of the first leaf pair responds similarly to growth temperature. When grown at 16°C, the first leaf pair was small and radial, growth at 22°C led to much longer, but still very narrow leaves, and growth at 29°C led to small but broad leaves. Note that the *rml1 *single mutant also showed enhanced root growth at the elevated temperature.

Growth of *bps1 rml1 *double mutants at 16°C led to production of a narrow radially-shaped first leaf pair that was much longer than the *bps1-2 *control (Figure 2). In *bps1 rml1 *double mutants grown at 22°C, the first leaf pair was long, but very narrow. Its narrowness was similar to the age-matched *bps1 *single mutant, but it was much longer. Finally, in *bps1 rml1 *double mutants grown at 29°C, the first leaf pair was flattened and had a distinct blade, very similar to the *bps1 *control. The similar effects of growth temperature on the first leaf pair of *bps1 *single and *bps1 rml1 *double mutants indicates that the shape of the first leaf pair is due to the *bps1 *mobile root-derived compound. This suggests that the first leaf pair of *bps1 rml1 *double mutants was exposed to the root-derived compound, while the later-arising rescued leaves were not.

### Competence to synthesize and respond to the *bps1 *signal is retained in older seedlings

Although the molecular target of the *bps1 *root-derived mobile compound is unknown, root-dependent arrest of early shoot development in *bps1 *seedlings indicates that the target is present at this early stage. However, we do not know if the molecular target is present later in development, nor do we know whether an older root retains the capacity to synthesize the mobile compound. To test this, we followed up on the observation that *rml1 *root development is rescued by supplying glutathione (GSH) [27]. We reasoned that supplying GSH to an older *bps1 rml1 *double mutant might restore growth of a *bps1*-like root. If this root retained the ability to synthesize and deliver the mobile compound, and its molecular target was present in older shoots, then we would expect to observe arrested leaf growth.

**Figure 3:**
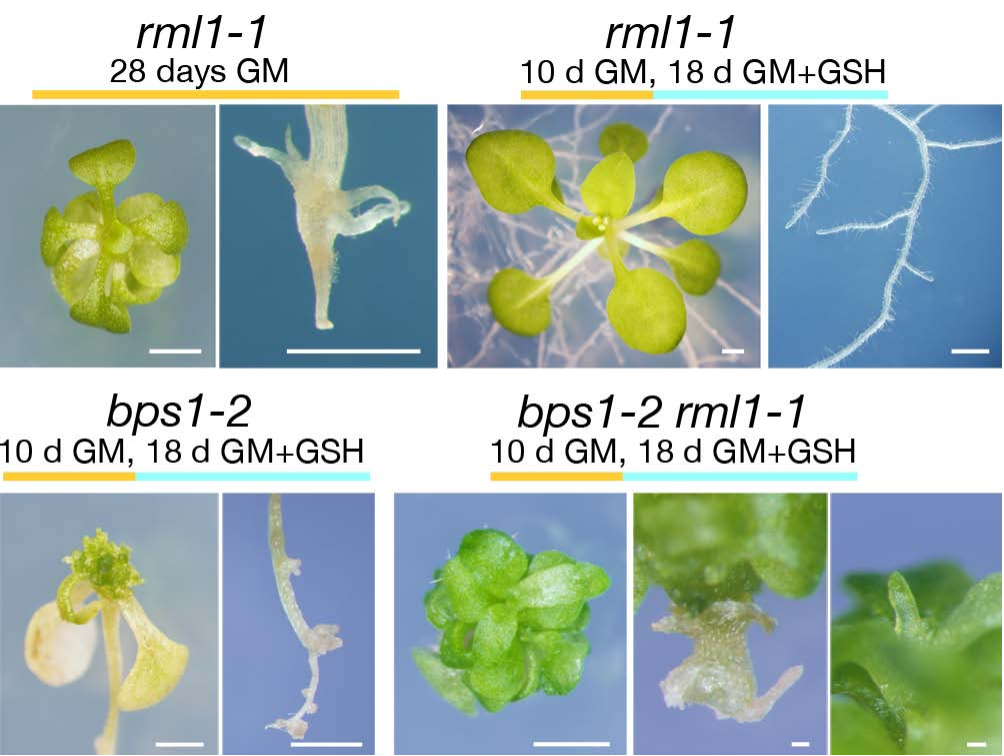
**Restored root growth in *bps1-2 rml1-1 *plants reinstates arrest of leaf development**. Top row: *rml1-1 *grown for 28 days on normal growth medium (GM) (left) and or transferred to GSH-supplemented GM at day 10 (right). The small stunted root is rescued by transfer to GSH (+). Bottom row: *bps1-2 *(left) and *bps1-2 rml1-1 *(right), both transferred from GM to GM+GHS at 10 days. The *bps1 *single mutants produced small roots and narrow radialized leaves. The *bps1-2 rml1-1 *double mutants showed a novel phenotype. The roots enlarged radially, they produced a few enlarged lateral-root-like organs, and arrested leaf primordia accumulated at the shoot apex. Size bars = 1 mm except for *bps1-2 rml1-1 *roots and leaf primordia, where bars = 0.1 mm.

Seeds segregating for both *bps1 *and *rml1-1 *were plated on standard growth media, and at 10 days, 90 seedlings with *rml1 *root phenotype were transferred to GSH-supplemented medium (approximately 22-23 were expected to be *bps1 rml1 *double mutants). At 18 days after transfer (28 total days), we analyzed their phenotypes. Most of the plants looked the same as *rml1-1 *controls; they produced normal-appearing roots and large flat leaves (Figure 3). However, 18 of the seedlings produced roots that were short, blunt, and very swollen, and had produced lateral roots somewhat similar to *bps1*. These plants also produced shoots with variable leaf shapes. Their first leaf pair was long and radial, the subsequently produced 5-9 leaves appeared flat (partially rescued), and with distinct petiole and blades. Finally, the newest arising leaves were short and radially shaped (*bps1-*like). This range of leaf phenotypes is consistent with restored synthesis and delivery of the *bps1 *mobile compound upon induction of root growth, and response in these later-arising vegetative leaves. These results indicate that roots retain the capacity to synthesize and deliver the *bps1 *mobile substance to the shoot, and that the shoots of older seedlings retain the ability to respond.

### The *bps1 *mobile compound: synthesis and delivery require neither the phloem nor endodermis

We next developed double mutants that combined *bps1 *with *altered phloem development *(*apl*), *shortroot *(*shr*), and *scarecrow *(*scr*) mutants. The *apl *mutant lacks phloem, *shr *lacks endodermis, and *scr *replaces endodermal and cortical cell layers with a single layer of mixed identity [29-33]. We predicted that if the phloem or endodermis were the sole site of synthesis of the *bps1 *mobile compound, or required for its transmission, then leaf development would be at least partially restored in the double mutants.

**Figure 4:**
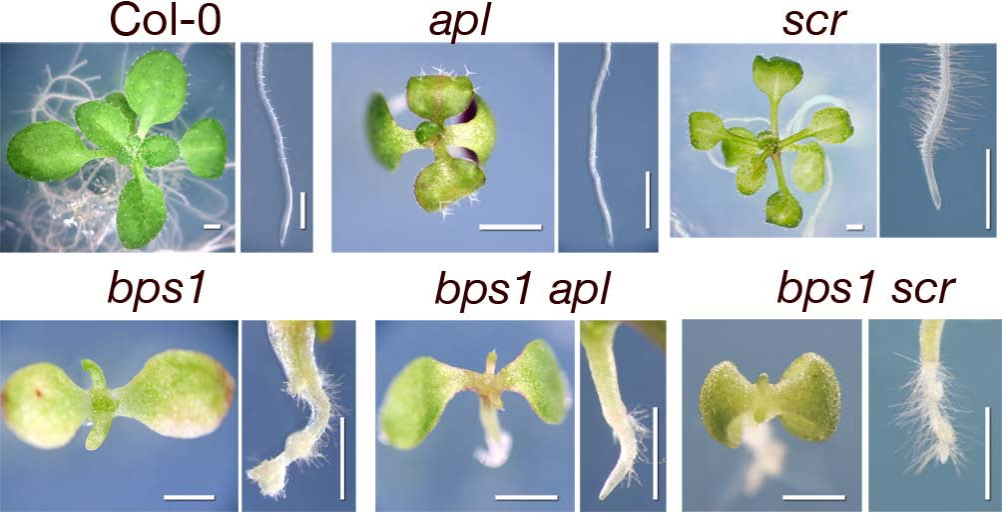
**Neither phloem nor endodermal cell types are required for production or transmission of the *bps1 *mobile compound**. Top row: shoot and root phenotypes of 15-day Col-0 (left), the phloem-deficient *apl *(middle), and *scarecrow *(*scr*, right). Bottom row: shoot and root phenotypes of *bps1 *(left), the *bps1 apl *double mutant (center), and *bps1 scr *double mutant (right). The double mutants show a leaf arrest phenotype that is similar to that of *bps1*, indicating that the root-derived mobile compound is still synthesized and transmitted to the shoot. Seedlings shown here were grown at 22°C and photographed at 15 days. Size bars: 1 mm.

The *bps1 apl *double mutants showed an arrested shoot phenotype that was indistinguishable from *bps1 *(Figure 4), indicating that phloem was dispensable for synthesis and delivery of the *bps1 *mobile compound, at least in these very small mutants. Similarly, both *shr bps1 *and *scr bps1 *double mutants resembled the *bps1 *single mutant (Figure 4, Table 3). Taken together, these data indicate that normal root development, including formation of the phloem and the endodermis, is not required for production and delivery of the *bps1 *signal.

**Table 3:** *shr *and *scr *radial patterning defects do not suppress the *bps1 *shoot phenotype

		Shoot Phenotypes Observed					
			
Plants analyzed	Total (n)	wild type (n)	*scr* (n)	*shr * (n)	*bps1* (n)	*Double mutant * (n)	χ^2^
*scr3-9 bps1-2 *F3	721	n/a	545	n/a	n/a	176	0.134^a^
*shr bps1-2 *F2	1040	576	n/a	194	270	n/a	0.528^b^
*shr*	167	129	n/a	38	n/a	n/a	0.449^a^
*bps1-2*	191	146	n/a	n/a	45	n/a	0.211^a^

n/a, not applicable

^a ^Critcal χ^2 ^values at 95% confidence is 3.841 when *df *= 1.

^b ^Critical χ^2 ^values at 95% confidence is 5.991 when *df *= 2.

### Shoot responses to the *bps1 *mobile root-derived compound

The reversible arrest of shoot development in *bps1 *mutants correlates with a loss of auxin responses [9], but the underlying mechanism of arrest is unknown. To broaden our understanding of shoot responses in *bps1*, we carried out a series of time-course analyses where we analyzed leaf size, shape, the distribution of dividing cells, and stress responses (necrotic lesion formation and appearance of ROS).

**Figure 5:**
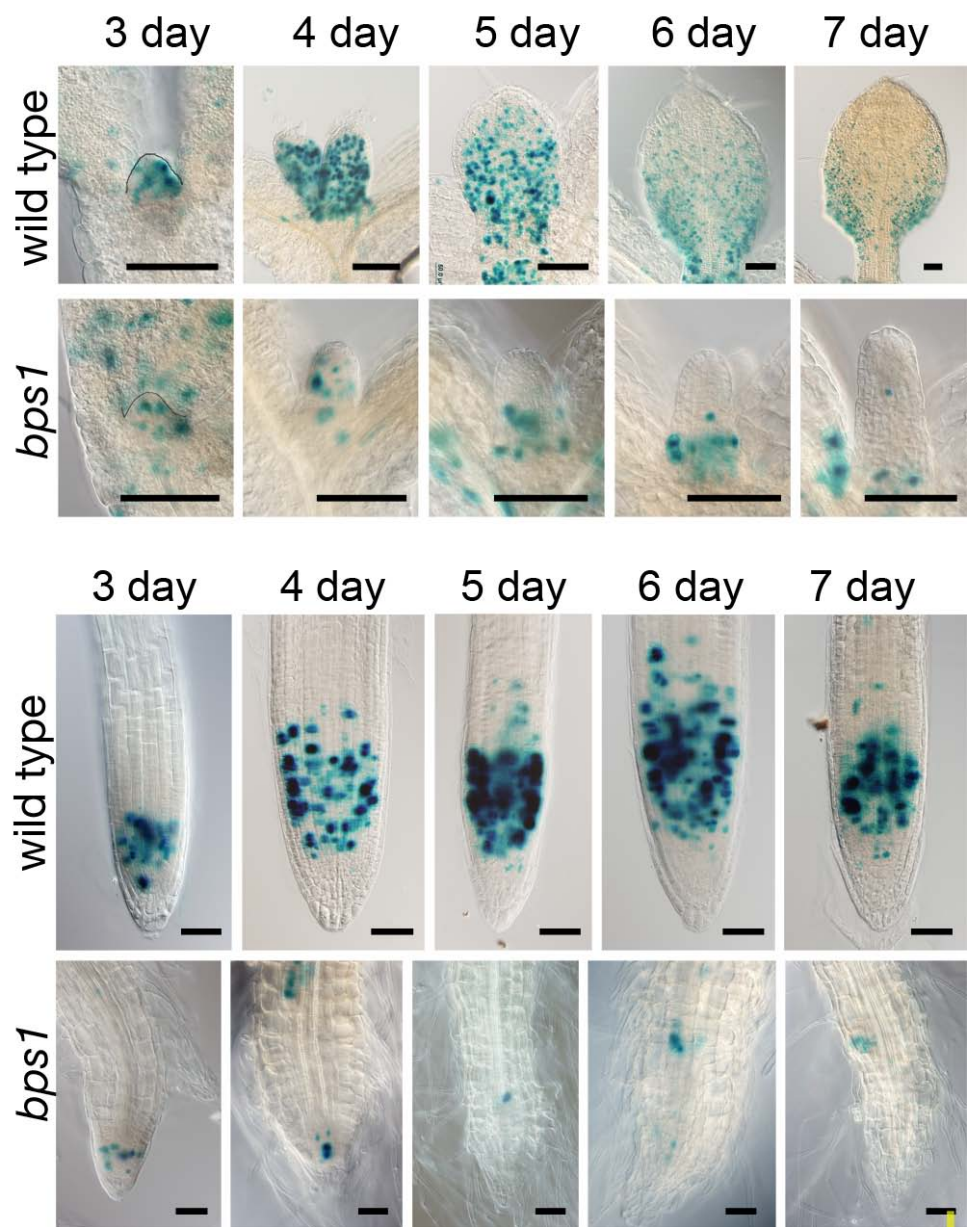
**Growth and cell cycle progress are diminished in *bps1 *leaves and roots**. GUS stained tissue from *bps1 *and wild-type plants carrying *CYCB1;1::GUS *transgene. Top set: representative leaves, harvested at the same time daily (days three through seven). Bottom set: roots from the same time points. For both organs, severe effects on growth and development were obvious in *bps1 *by day four, and included dramatic reduction in the number of G2/M phase cells. Size bar = 0.1 mm (leaves), and 0.05 mm (roots).

Dividing cells were identified using the *CYCB1;1::GUS *reporter, a cell cycle reporter expressed in cells at the G2/M phase [34]. Patterns of *CYCB1;1::GUS *expression in Arabidopsis are well characterized; early leaf development shows a nearly uniform distribution of GUS-staining (i.e. dividing) cells, while later in development cell divisions become restricted to the leaf base and provascular tissue [35] (Figure 5). In *bps1 *mutants, the three-day leaf primordia largely matched wild type in terms of size, shape and *CYCB1;1::GUS *expression patterns, however there were pronounced differences by day four. The four-day wild type leaf was much larger than that of *bps1 *and *CYCB1;1::GUS*-staining cells were distributed throughout, while the small four-day *bps1-2 *leaf had only a few *CYCB1;1::GUS*-staining cells. At day five, the wild-type leaf started to show distinct lamina expansion, and a slight tendency for there to be more GUS-staining cells toward its proximal end. By contrast, the five-day *bps1 *leaf showed no sign of lamina expansion, and few *CYCB1;1::GUS*-staining cells. The six-day wild-type leaf showed a strong reduction of *CYCB1;1::GUS*-staining cells at the distal end, and was much larger than the five-day wild-type leaf, while the corresponding *bps1 *leaf was largely unchanged. By day seven, the wild-type leaf was even larger and the few *CYCB1;1::GUS *expressing cells were at the leaf base. Similarly, the *bps1 *seven-day leaf had only a few *CYCB1;1::GUS*-staining cells, and most were restricted to the leaf base. This analysis revealed a fully penetrant leaf arrest phenotype. In addition, despite the striking reduction in numbers of dividing cells, the apical/basal spatial control of cell divisions appeared to be intact.

While carrying out this analysis of leaf development, we also compared patterns of *CYCB1;1::GUS*-staining in roots (Figure 5). In the wild type, *CYCB1;1::GUS*-staining patterns were restricted to the root meristem, as has been described previously [36], and a similar pattern was observed between days three and seven. By contrast, at all time points, the *bps1 *root had fewer *CYCB1;1::GUS*-staining cells.

**Figure 6:**
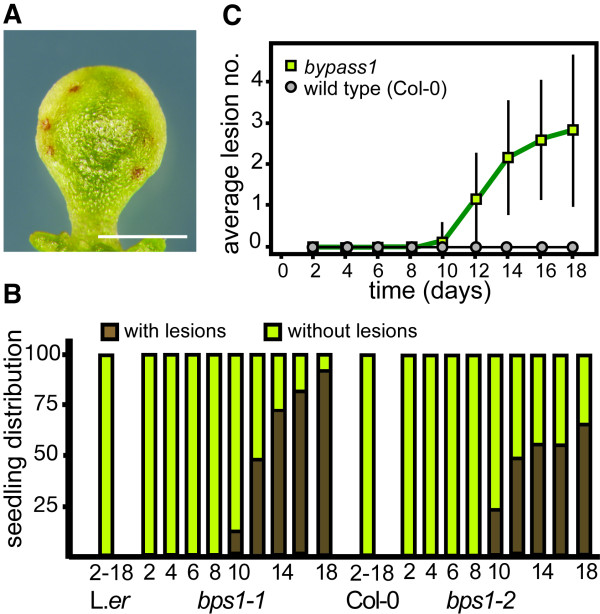
**Necrotic Lesion formation is a late and not fully penetrant phenotype in *bps1 *mutants**. (A). 14-day *bps1-1 *cotyledon with necrotic lesions. (B). Onset and penetrance of necrotic lesion formation. Green bars represent the percent of plants with no lesions, and brown bars represent the percent with one or more necrotic lesion. Neither the L.*er *nor *Col-0 *wild type produced any necrotic lesions, whereas for both *bps1-1 *and *bps1-2 *mutants formed necrotic lesion starting between day 8 and 10, after which lesion number increased steadily. N = 84 (L.*er*), 140 (*bps1-1*), 48 (Col-0), 80 (*bps1-2*) (C) Average number of lesions per seedling. The number of lesions per seedling is depicted as a function of time, and bars show standard deviation. size bar = 1 mm.

The *bps1 *mutant analysis revealed occasional necrotic lesions on *bps1 *cotyledons (Figure 6A). To assess a possible relationship between these lesions and leaf arrest, we carried out another time-course analysis, this time examining wild type and *bps1 *mutants for necrotic lesion formation. In *bps1 *mutants, necrotic lesions began to appear between 8 and 10 days. They were restricted to cotyledons, and never observed on leaves, hypocotyls, or roots, and necrotic lesions were never observed on the wild type (Col-0 or L.*er*) (Figure 6B). More *bps1-1 *seedlings formed necrotic lesions than *bps1-2*, and by day 18, 92% of the *bps1-1 *plants had at least one necrotic lesion. The average lesion number per plant was highly variable, and increased over time (Figure 6C); by day 18 the *bps1-2 *mutants had between zero and seven necrotic lesions. Both *bps1-1 *and *bps1-2 *are null alleles [1], and so we attribute the difference in lesion formation to their genetic backgrounds (Col-0 for *bps1-2 *and L.*er *for *bps1-1*).

**Figure 7:**
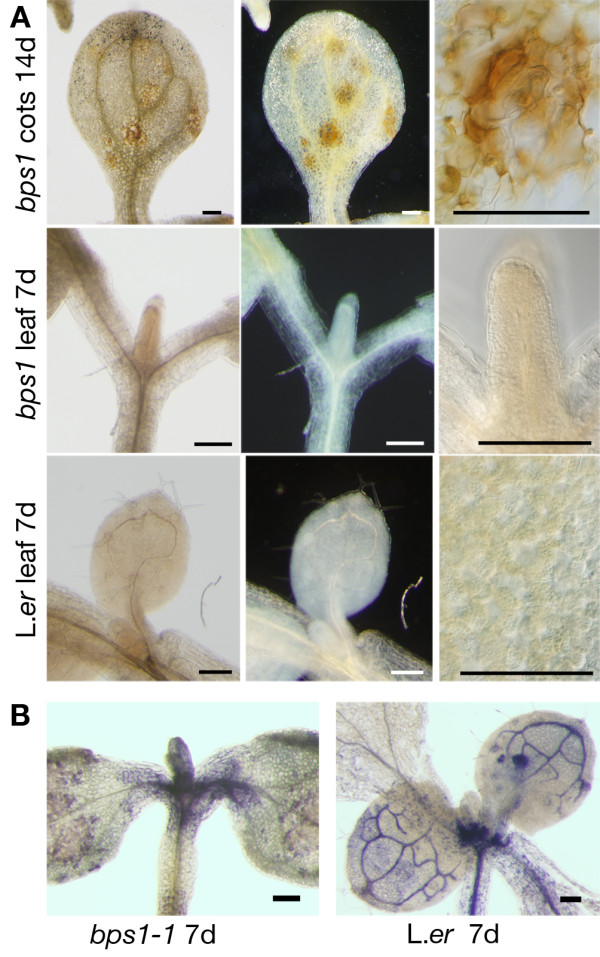
**ROS is not increased in arrested *bps1 *leaves**. (A). Hydrogen peroxide, visualized using DAB, was found in necrotic lesions and associated with vascular tissue, but it was not elevated in the *bps1 *leaf. (B) Superoxide, visualized using NBT staining, was found associated with vascular tissue, but it was not elevated in the *bps1 *leaf. Bars = 200 μm.

Because lesion formation is typically preceded by reactive oxygen species (ROS) [37-40], we compared ROS in *bps1 *and wild type shoots using diaminobenzadine (DAB) to assay for hydrogen peroxide (H_2_O_2_) and nitroblue tetrazolium (NBT) to assay for superoxide. Because the *bps1-1 *allele showed a more robust necrotic lesion phenotype, these analyses used *bps1-1 *and L. *er*. Both staining procedures produced a strong reaction in the vascular tissue, consistent with a role for ROS in lignification [41,42]. We found H_2_O_2 _in the 14-day *bps1 *cotyledons, typically in positions surrounding the developing necrotic lesions (Figure 7A), but did not observe any nonvascular staining in the wild type cotyledon (data not shown). Additionally, we did not detect H_2_O_2 _in *bps1 *leaves. Similarly, superoxide was primarily associated with vascular tissue in wild type leaves, and it was nearly absent from the leaves of *bps1 *mutants (Figure 7B). Because accumulation patterns of these two ROS were similar for the wild type and *bps1 *mutants, severe oxidative stress does not appear to cause the *bps1 *leaf developmental arrest.

## Discussion

Physiological studies have implicated long distance signaling as a link between the development and physiology of roots and shoots [20]. However, only a small number of long-distance signaling pathways have been verified molecularly. In Arabidopsis *bps1 *mutants, the non-cell-autonomous activity of mutant roots suggests that BPS1 might function to limit the synthesis of a root-derived mobile signaling molecule [9].

### Capacity to Synthesize the BPS1 Mobile Compound

A central feature of *bps1 *mutants is that the root is the source of a biologically active mobile compound, which we refer to as the *bps1 *signal. Here, we extended our understanding of the conditions under which the mutant root produces this compound. Previously we showed that cutting off the root led to rescue of the first leaf pair [9]. Indeed, we have now found that arresting post-embryonic *bps1 *root growth also resulted in rescue of leaf development. However, in contrast to root excision, the first leaf pair was only mildly rescued, and strong rescue was delayed until leaf three. These observations indicate that the *bps1 *root, despite post-embryonic arrest, retained a transient ability to supply the *bps1 *signal to the shoot.

We used two related approaches to arrest post-embryonic root growth: we caused arrest through the depletion of GSH either genetically (using the *rml1-1 *mutant) or chemically (using BSO). In both cases, the first leaf pair in GSH-depleted *bps1 *was larger than that of untreated *bps1 *mutants, and the first leaf pair of *bps1 rml1-1 *double mutants were consistently larger than that of BSO-grown *bps1*. Here, a larger leaf size probably reflects an earlier block to GSH synthesis in the mutant, and therefore an earlier reduction in *bps1 *signal synthesis.

Similarly, we found that restoring development of *bps1 *roots (by GSH provision to *bps1 rml1-1 *seedlings) reinstated arrest of leaf development. The extended capacity to produce and respond to the mobile compound is in line with physiological studies of drought-evoked long distance signaling, which has been documented in diverse plants, and at varying developmental stages [4].

A possibly less obvious question is why growth-arrested roots (i.e. *bps1 rml1-1 *double mutants and BSO-grown seedlings) show a decreased ability to arrest shoot growth. One possibility is that *bps1 *signal synthesis has a direct requirement for GSH. Alternatively, either synthesis or transmission to the shoot requires active root growth and cell division.

The maintenance of shoot arrest in *apl bps1 *double mutants is consistent with a link to root growth. Although *apl *mutants have determinate roots [29], growth ceases later than for *rml1-1 *or BSO-treated plants, and the *apl bps1 *analysis was carried out prior to evidence of root cell division arrest. However, if root growth is a requirement for *bps1 *signal synthesis, then we would need to be able to explain constitutive synthesis of *bps1 *signal in *bps1 *mutants, which show primary root arrest soon after germination. One possibility is that synthesis is sustained by lateral roots, which initiate repeatedly. Alternatively, *bps1 *roots (including the primary) expand radially, and this radial growth might also sustain synthesis of the *bps1 *signal.

### *bps1 *signal transmission

Movement of the *bps1 *signal from the root to the shoot is likely to use the plant's vascular system. Two vascular tissues are specialized for long-distance movement: the phloem, which transports photosynthate, and also mRNAs and proteins; and the xylem, which primarily transports water and dissolved nutrients. Here, we found that the shoot undergoes arrest in *bps1 apl *double mutants, which lack phloem [29]. The simplest conclusion is that the *bps1 *signal moves in the xylem. However, this conclusion is not definitive, because the very small size of *bps1 apl *double mutants doesn't preclude movement by diffusion.

### Shoot responses to the mobile *bps1 *signal

The small leaf size and reduced number of *CYCB1;1::GUS *expressing cells are a fully penetrant *bps1 *phenotype. Strikingly, although reduced in number, the pattern of *CYCB1;1::GUS-*expressing cells mimicked the wild type pattern: leaf primordia showed an even distribution of diving cells, but as the mutant leaves matured, dividing cells were restricted to the base of the leaf. The retention of a normal pattern of dividing cells shows that some aspects of leaf developmental programming persist in *bps1 *mutants. This result hints that instead of altering development, the *bps1 *signal might instead disrupt the link between development and cell cycle control.

Another phenotype in *bps1 *mutants is the formation of necrotic lesions. These were late-appearing and not fully penetrant. Necrotic lesions have been observed in a wide range of Arabidopsis mutants. These include plants with defects in syntaxin genes [43], and mutants with defects in the cytochrome P450 gene *CYP83B1*, which results in excess auxin synthesis [44]. Necrosis is typically associated with plant defense responses, and can be a secondary consequence of elevated expression of defense genes, such as observed in the developmental mutant *asymmetric leaf 1 *[45] and in response to phosphate deficiency [46,47].

## Conclusions

The results presented here support the phenomenon of shoot arrest by a root-derived molecule in *bps1 *mutants. A key question raised by discovery and characterization of this mutant is whether the *bps1 *mutation exposes a novel root-to-shoot signaling molecule or a metabolic intermediate with toxic effects on shoot development. The crucial difference between these two concepts is that a novel root-to-shoot signaling molecule would be present in the wild type, while a metabolic intermediate would only accumulate in *bps1 *mutants. Because the synthesis of the root-derived molecule requires post-embryonic root development and aerial organs appear to arrest growth prior to showing any signs of toxicity (necrosis), we tend to favor the hypothesis that *bps1 *reveals a novel root-to-shoot signaling pathway. A full resolution of this issue awaits biochemical identification of this mobile molecule. Regardless of the nature of the root-derived compound, it should be pointed out that under either scenario the *bps1 *mutation has unveiled a molecule with potent biological activity. Despite the impact of root-to-shoot communication on plant productivity, the molecular mechanisms involved are poorly understood. The *bps1 *mutation could be utilized as a tool to begin to dig into the pathways that both synthesize and respond to root-derived growth modulators.

## Methods

### Plant Growth

All seeds were cold-shocked for 2-4 days in darkness at 4°C, and most grown in 24 hour light at the 22°C, unless noted otherwise. Growth media composition is 0.5X MS salts (Caisson labs), 1% sucrose, 0.5g/l MES, pH 5.8, 0.8% phytoblend agar (Caisson Laboratories). Seedlings were grown in Conviron TC30 growth chambers under light and temperature regimes as described.

### Plant Materials

Mutant alleles used: *bps1-*2 (Col), *bps1-1 *(L.*er*), *rml1-1 *(Col, received from Z.R. Sung), *scr-3 *(Col, CS3997), *shr *(Col, SALK_002744), *apl *(Col, received from M. Bonke), and CYCB1;1::GUS seeds were received from J.L. Celenza.

### GUS Staining

The CYCB1;1::GUS transgene was crossed into both *bps1-1 *and *bps1-2*, and F3 lines homozygous for the transgene and segregating for *bps1 *were identified. We plated these lines (and control wild-type transgenic) on normal growth media, and subjected them to a 2-7 day cold shock (4°C). Each day, plates were transferred to a 22°C growth chamber. GUS staining followed previously published protocols [48].

### Conditional Root Arrest

Arrest of roots using BSO was carried out by making our standard GM (above), and supplementing it to 2.5 mM BSO (DL-Buthionine-[S,R]-sulfoximine, Sigma), and *bps1-2 rml1-1 *double mutants were generated by standard methods. For both BSO and *rml1 *experiments, plants were grown in short day (8 hours light/16 hours dark) at 22°C. To reinstate root growth of *rml1-1*, we supplemented the media to 750 μM glutathione (Acros Organics) [27]. To test whether the number of plants producing broad leaves upon root excision in the presence of GSH was statistically different from that observed in the absence of GSH (Table 2), we performed hypothesis testing for proportions using the Z-score method. If GSH were the root-shoot signal, we would predict that the number of plants forming broad leaves under GSH+ conditions would be less than under GSH-conditions (the null hypothesis). The statistical tests indicate that the number of plants producing leaves in the presence of GSH is not less than or equal to the number producing leaves in the absence of GSH. Because the calculated P value is low, we must reject the null hypothesis in support of the alternative hypothesis that the number of plants producing leaves under GSH+ conditions is greater than GSH-conditions. This indicated that the root-to-shoot signal is not GSH.

### Stress symptom analyses

Necrotic lesion formation was assessed by a visual inspection of *bps1 *and wild type seedlings. All organs of the investigated seedlings were examined on alternate days. To visualize patterns of H_2_O_2 _in seedlings (wild type and *bps1*), we used 3,3'-diaminobenzidine (DAB) staining as described [49,50]. We infiltrated 0.1% (W/V) DAB (Sigma), pH3.8, and allowed staining to progress for 4-6 hours. After staining, samples were cleared in 70% ethanol, and then transferred to 40% w/v glycerol, mounted on glass slides, and examined on Olympus BX50 and Olympus SZX16 microscopes. Visualization of superoxide patterns used nitroblue tetrazolium staining protocols as described [51,52].

## Authors' contributions

JMVN carried out *rml1 *and BSO root arrest experiments and the *apl bps1*, *shr bps1*, and *scr bps1 *double mutant analyses. CM carried out analyses of ROS, characterized the lesion formation phenotype, and analyzed shoot phenotypes following resumption of root development. LES carried out the pCYCB1;1::GUS analyses. LES and JMVN planned the project together, and the manuscript was primarily written by LES with assistance from JMVN. All authors read and approved the final manuscript.
